# The acute effect of incorporating lettuce or watercress into a moderately high-fat meal on postprandial lipid, glycemic response, and plasma inflammatory cytokines in healthy young men: a randomized crossover trial

**DOI:** 10.1186/s12944-021-01487-9

**Published:** 2021-07-15

**Authors:** Sahar Shokraei, Nafiseh Khandouzi, Zahra Sina, Javad Nasrollahzadeh

**Affiliations:** Department of Clinical Nutrition & Dietetics, National Nutrition and Food Technology Research Institute, Faculty of Nutrition Sciences and Food Technology, Shahid Beheshti University of Medical Sciences, 46, Hafezi St., Farahzadi Blvd., Shahrak Qods, P.O.Box: 19395-4741, Tehran, Iran

**Keywords:** Vegetable, Lettuce, Watercress, Postprandial, Lipemia, Glycemic, Inflammatory markers

## Abstract

**Background:**

Postprandial responses to food mostly depend on the composition of the meal and the consumption of vegetables may modulate this postprandial state. In this study, the effects of lettuce or watercress consumption with a moderately high-fat meal (40% kcal from fat) on postprandial lipemia, glycemia, and inflammatory cytokines were determined in healthy men.

**Methods:**

This randomized, 3-arm, crossover study was conducted in sixteen healthy young men with a mean ± SEM age and body mass index (in kg/m2) of 22.8 ± 0.5 years old and 23.7 ± 1.16, respectively. Lettuce and watercress were added to the test meal in portions of 100 g and cellulose was added to the control meal. Thereafter, blood samples were collected by passing 0, 1, 2, 3, and 4 h for analysis. The postprandial response was measured in plasma glucose, triglycerides (TG), total cholesterol, high-density-lipoproteins cholesterol (HDL-C), and low-density-lipoproteins cholesterol (LDL-C), as the area under the postprandial curve (AUC). Moreover, plasma tumor necrosis factor-α (TNF-α) and interleukin-6 (IL-6) were determined once before and once 4 h after the meal’s consumption.

**Results:**

The 0–4-h AUCs for glucose (385.7, 361.9, and 382.3 mg/dL for the control, lettuce, and watercress meals, respectively) were calculated to be lower when meal was consumed with lettuce compared to the control (*P* < 0.05) and watercress (*P* < 0.01) meals. The maximum values of insulin were obtained as 43.8 ± 18.8, 33.5 ± 19.5, and 42.8 ± 17.7 μIU/mL for the control, lettuce, and watercress meals, respectively. As well, the lettuce-containing meal more reduced the AUC for insulin compared with the control (*P* < 0.05), but not watercress. Notably, plasma TG, total cholesterol, HDL-C, and LDL-C had no significant differences among the meals. Moreover, the levels of plasma IL-6 and TNF-α did not differ among the meals.

**Conclusion:**

In this study on healthy men, the addition of lettuce to a moderately high-fat meal delayed the postprandial glycemic response. However, the effect of the consumption of these vegetables on postprandial responses in subjects with cardiometabolic risk factors remains to be elucidated yet.

This clinical trial was registered at the Iran Clinical Trials Registration Office (IRCT) on March 3, 2018, with an ID of IRCT20160702028742N4 (https://www.irct.ir/user/trial/23233/view).

## Introduction

Increasing evidences suggest that the postprandial state, characterized by lipemia and glycemia/insulinemia, can be considered as a contributing factor to the development of chronic metabolic diseases [1–3]. A higher postprandial triglycerides (TG) concentration, particularly after 2 to 4 h from eating a meal, was also found to be associated with cardiovascular events more closely than fasting TG levels [4]. Given its potential association with the incidence of cardiovascular diseases, postprandial TG has been incorporated into some clinical guidelines [5]. Similarly, postprandial blood glucose, but not fasting levels, was shown to be an independent risk factor for cardiovascular events in those with type 2 diabetes [6]. Since human beings are predominantly exposed to postprandial lipemia and glycemia throughout the day, so a good regulation of the postprandial state can potentially be effective on preventing cardiometabolic changes.

It was indicated that meal composition affects both the postprandial glycemia and lipemia [7, 8], as well as a postprandial inflammatory response [9, 10]. Vegetables are rich in dietary fiber and various micronutrients, so their consumption along with meals has the potential of improving postprandial responses by blunting the postprandial increase in glucose, TG, and inflammation [11]. In this regard, lettuce (*Lactuca sativa* L.), known as a healthy food, is one of the most popular and widely consumed vegetables worldwide. Although less studied, lettuce has been shown to exert a beneficial effect on lipid metabolism and tissue oxidation in previous preclinical studies [12]. Accordingly, the healthy properties are attributed to its fiber moiety, but lettuce also contains large amounts of water-soluble antioxidants such as vitamin C and various polyphenols, as well as lipid-soluble antioxidants such as lutein and tocopherols [12–15]. Of note, watercress (Nasturtium officinalis) is known as a rich source of polyphenols [16], so it is increasingly being added to dishes due to having valuable health benefits and a rich chemical composition [17, 18]. Watercress has been extensively studied under both in vitro and in vivo conditions in terms of its chemopreventive properties, whereas little attention has been paid to its potentially beneficial metabolic properties. In some previous experimental studies, lettuce and watercress have been shown to improve multiple cardiovascular risk factors [12, 19, 20]. However, limited information still is available on the acute postprandial effects of these vegetables on glycemia, lipidemia, and inflammatory mediators. Therefore, the present study was designed to investigate the effects of the addition of either lettuce or watercress to a moderately high-fat, fast food style meal on postprandial lipids, glucose, insulin, and inflammatory cytokines.

## Methods

### Participants

A group consisting of 16 male healthy volunteers aged between 20 and 30 years old were included in this study. The participants were the volunteer students from the local medical school. Subsequently, they were randomly assigned into either one of three parallel treatments. The order of the three test meals (the control meal, romaine lettuce with the meal, and watercress with the meal) was randomly determined prior to the beginning of the study. All the participants received all these three treatments. The exclusion criteria included body mass index (BMI) < 18.5 or > 27 kg/m^2^, past history or the current diabetes, hypertension or the use of anti-inflammatory medications or supplements (e.g., fish oil).

The present study protocol was approved by the Ethics Committee of the National Nutrition and Food Technology Research Institute of Iran. Written consent was also obtained from each participant prior to participation in this study, after the time that the purposes of the study as well as their right to withdraw from the study at any time were explained to them. This study was conducted in terms of the principles outlined in the Declaration of Helsinki and was registered at the Iranian Registry of Clinical Trial (IRCT) with an ID number of IRCT20160702028742N4.

### Experimental design

This was a randomized, placebo-controlled, 3-period crossover study in which the included subjects completed 3 meal challenges consisting of single-day visits, each one lasted for ∼4 h. At least 1 week interval was allowed between each one of the study days. Thereafter, the participants were instructed to avoid any anti-inflammatory, dietary or herbal supplements such as fish oil and ginger 24 h prior to each test day. The volunteers were also advised to maintain the same dietary habits and physical activity level until the completion of the three testing sessions of the current study. In addition, the subjects were instructed to consume dinner with a similar composition at the night before each test day.

After a 12-h fasting, the participants arrived to the research unit of the nutrition clinic in the Faculty of Nutrition Sciences and Food Technology. Immediately at the time of arrival, a fasted blood draw was taken for plasma via venepuncture. The participants were given a moderately high-fat meal within 20 min. As well, the venous blood samples were collected at 1, 2, 3, and 4 h in sodium citrate tubes, and plasma was isolated from each sample by centrifugation. All the plasma samples were stored at − 80 °C until further analyses. Throughout the test period, the participants were not allowed to consume any food, but they could drink bottled water ad libitum. Notably, the participants either stayed at the research center or they were left between blood draws to attend their classes.

### Test meals

In each one of the meal interventions, a similar fast food style meal was consumed, which was comprised of white bread, hamburger, cheese, butter, and mayonnaise, and the test vegetables were also added in raw form to the meal. The prepared meals were served with 120 mL of a diet drink. This study included two vegetables (100 g each): romaine lettuce and watercress, and one control meal containing no vegetable. Correspondingly, in the control meal, a powder containing 2.4 g of microcrystalline cellulose was suspended in the diet drink, which was then consumed with the meal. All the meal components were weighed before the preparation process. At each test day, all the meal components were freshly prepared and also weighed before the meal preparation. At each meal challenge, the dishes were mixed in order to be consumed during 15 min in total. The nutritional composition of the meals based on the results obtained from food databases is summarized in Table 1.

**Table 1:** Composition of test meals

	Control	Lettuce	Watercress
**Energy (Kcal)**	760	778	763
**Carbohydrate (g)**	94.3	97.8	94.7
**Carbohydrate (%)**	49.7	50.3	49.7
**Protein (g)**	19.0	20.1	19.5
**Protein (%)**	10	10.3	10.2
**Fat (g)**	34	34	34
**Fat (%)**	40.3	39.4	40.1
**Saturated Fat (g)**	15.9	15.9	15.9
**Monounsaturated fat (%)**	11.6	11.3	11.5
**Polyunsaturated fat (%)**	10.6	10.4	10.6
**Cholesterol (mg)**	87	87	87
**Vegetable(g)**	–	100	100
**Cellulose (g)**	2.4	–	–
**Dietary fiber (g)**	2.6	2.6	0.7

### Biochemistry analysis

Glucose and TG, total cholesterol, high-density-lipoproteins cholesterol (HDL-C), and low-density-lipoproteins cholesterol (LDL-C) were analyzed in batches with an autoanalyzer Selectra 2 (Vital Scientific, Spankeren, The Netherlands), using some commercial kits (Pars Azmoon, Karaj, Iran). Additionally, enzyme-linked immunosorbent assay kits were used to measure plasma insulin (Diametra, Perugia, Italy), interleukin 6 (IL-6), and tumor necrosis factor alpha (TNF-α) (eBioscience, San Diego, USA) in terms of the manufacturer’s instructions.

### Statistical methods

The primary outcome measure was considered as a change in plasma insulin area under the curve (AUC). A priori sample size calculation was performed using G*Power 3.1 software [21]. It was indicated that for a significance level of α = 0·05, including 16 subjects will provide 80% power to detect an effect size of 1.03 among the means used for the primary endpoints of insulin AUC. In the current study, because of the non-existence of a prior clinical trial with a similar intervention, the effect size was calculated using the results of a previous study. In this study, a vegetable from the brassica family (Bok choy) was added to the test meal of the healthy adults, which decreased the AUC 120 min for insulin from 2358**.**2 ± 687.6 to 1744**.**7 ± 480.1(μU*min/mL) [22] .

The normality of the parameters’ distribution was tested using the Shapiro–Wilks test. The obtained results were reported as mean ± standard error of the mean (SEM), otherwise they were stated. At the next stage, AUCs for glucose, insulin, TG and total cholesterol, HDL-C, and LDL-C were calculated by applying the trapezoidal rule. Subsequently, all the AUCs were calculated using a computer spreadsheet (Microsoft Office Excel 2010). Moreover, the differences in postprandial insulin, glucose, and TG and cholesterol responses were evaluated using the total AUC (0–4 h). Due to the crossover design of this study, between-treatment comparisons of AUC were done using analysis of variance (ANOVA) for repeated measures by considering food as within-subject factor followed by the post hoc Bonferroni’s test. Postprandial changes in plasma glucose, insulin, lipids, and cytokines were compared using a repeated-measures ANOVA by considering both treatment and time as within-subject factors. Accordingly, the main effects of treatment and time, as well as the time × treatment interaction, were investigated, and post hoc comparisons were then carried out using Bonferroni correction for multiple comparisons. All the analyses were performed using SPSS Statistics ver. 22 (IBM Corporation).

## Results

**Fig. 1:**
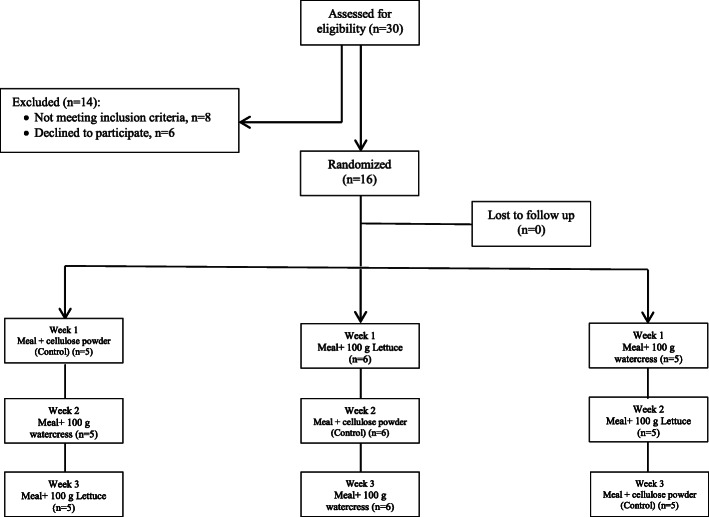
Flow chart of the participants

The characteristics of the participants who completed the study, are presented in Table 2. It is noteworthy that all the subjects completed the study (Fig. 1.) and consumed entire test meals. However, the meal with lettuce was more acceptable than with the meal with watercress. Regarding the pre-meal baseline characteristics of the participants, values had no differences, but in the control compared with lettuce meal visits, a tendency towards lower levels of insulin was found (*P* = 0.09). During the study period, the body weights of the participants did not change significantly.

**Table 2:** Characteristics of participants who completed the study

	Men (*n* = 16)
Age, y	22.8 ± 0.5
Weight, kg	73.69 ± 3.88
BMI, kg/m^2^	23.60 ± 1.16
Glucose, mg/dL	93.50 ± 1.62
Total cholesterol, mg/dL	141.25 ± 6.05
HDL-C, mg/dL	42.23 ± 2.32
LDL-C, mg/dL	79.08 ± 4.79
TG, mg/dL	77.37 ± 10.99

Values are means ± standard errors

*BMI* Body mass index, *HDL-C* High-density-lipoproteins cholesterol, *LDL-C* Low-density-lipoproteins cholesterol, *TG* Triglycerides

### Lipids

**Fig. 2:**
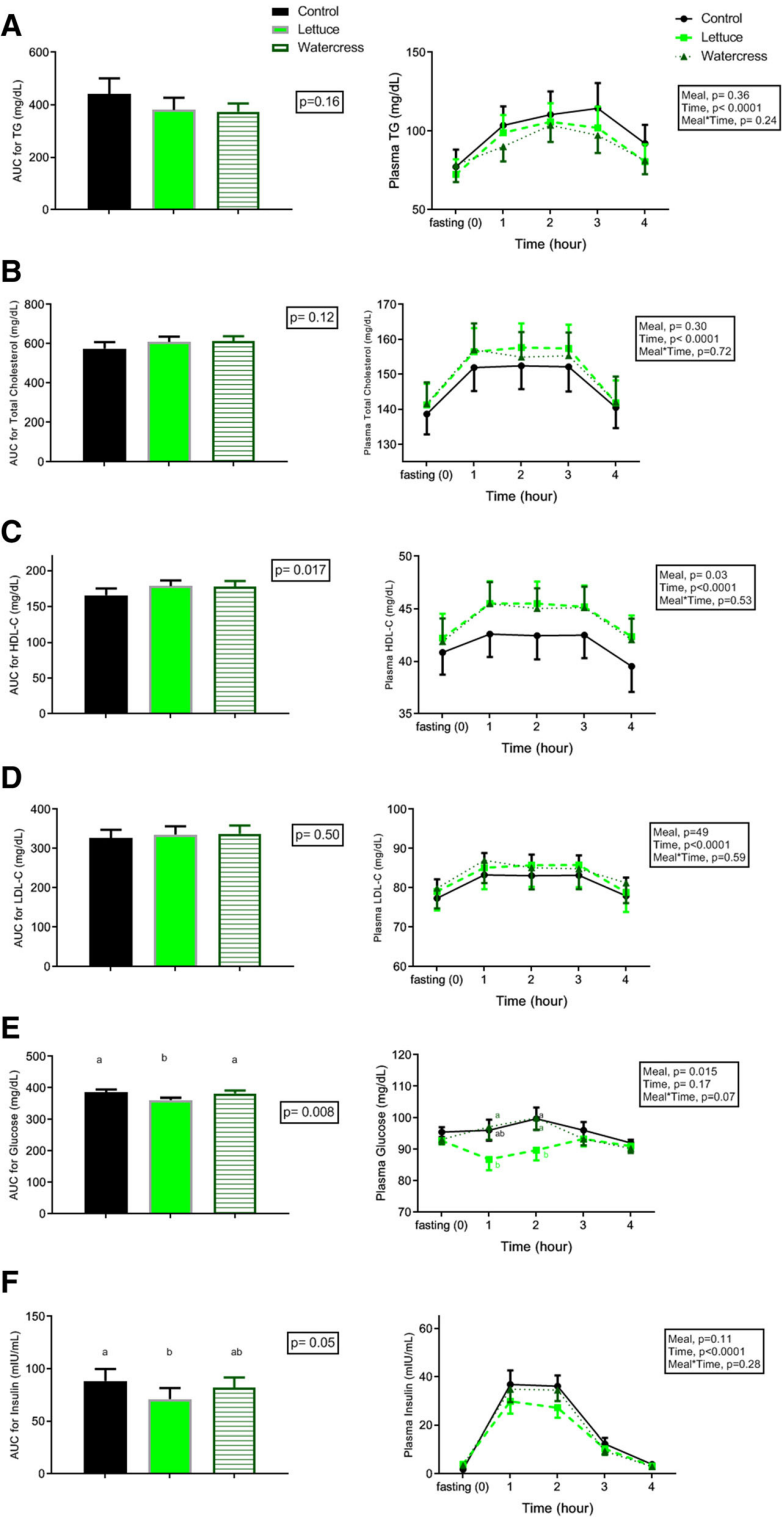
Plasma glucose, insulin, and lipid responses after the consumption of control, lettuce or watercress meals in healthy subjects. Left: AUC between 0 and 4 h. Right: Postprandial responses at different time points. Values are presented as means with their standard errors (*n* = 16). Statistical analysis was assessed by repeated-measure ANOVA. AUC, area under the curve; HDL-C, high-density lipoprotein cholesterol; LDL-C, low-density lipoprotein cholesterol; and TG, triglycerides. Labeled values without a common letter indicate difference between the three treatments, *P* < 0.05

As shown in Fig. 2-A, postprandial plasma TG, total cholesterol, HDL-C, and LDL-C significantly increased after the consumption of three meals (*P* < 0.0001), but these did not differ among the three interventions. The TG response was found at the highest level by passing 3 h from the control meal, whereas by consuming lettuce or watercress with the meal, it peaked up at 2 h post-meal and decreased thereafter. However, the maximum value of TG or the difference between the baseline and maximum values were not significant among the three treatments (Table 3). In addition, the AUC values for postprandial TG, total cholesterol, and LDL-C responses were not significantly different among the three studied meals. Regarding AUC for HDL-C, although the repeated measures ANOVA revealed a *P* = 0.017, Bonferroni pairwise comparisons test showed *P* = 0.06 when comparing control with lettuce, and *p* = 0.07 when comparing control with watercress. Thus, AUC for HDL-C tended to be higher after the consumption of lettuce or watercress.

**Table 3:** Maximum value of plasma lipid and glycemic markers after the consumption of control, lettuce or watercress meals in healthy subjects

	Control	Lettuce	Watercress	*P* value^1^
**TG** (mg/dL)				
Max	121.8 ± 15.4	111.3 ± 13.2	108.2 ± 12.1	0.320
Max-baseline difference	44.5 ± 8.1	38.8 ± 5.2	29.6 ± 5.3	0.196
**Total cholesterol** (mg/dL)				
Max	155.8 ± 6.9	160.9 ± 6.8	161.3 ± 7.1	0.146
Max-baseline difference	17.2 ± 2.0	19.7 ± 2.1	19.7 ± 3.2	0.619
**HDL-C** (mg/dL)				
Max	43.7 ± 2.2	44.2 ± 3.2	46.4 ± 2.0	0.380
Max-baseline difference	2.8 ± 0.5	1.9 ± 2.6	4.5 ± 0.7	0.436
**LDL-C** (mg/dL)				
Max	85.1 ± 5.4	87.8 ± 5.6	88.7 ± 5.5	0.309
Max-baseline difference	7.7 ± 1.2	8.7 ± 1.5	8.8 ± 1.5	0.773
**Glucose** (mg/dL)				
Max	105.1 ± 2.9	100.2 ± 2.1	101.7 ± 6.9	0.559
Max-baseline difference	9.5 ± 1.9	7.4 ± 2.0	8.5 ± 6.7	0.802
**Insulin** (μIU/mL)				
Max	43.8 ± 4.7 ^a^	33.5 ± 4.9 ^b^	42.8 ± .4 ^ab^	0.038
Max-baseline difference	41.9 ± 4.4 ^a^	29.5 ± 4.3 ^b^	39.3 ± 3.9 ^ab^	0.019

Values are means ± standard errors

^1^ Data were analyzed by repeated-measure ANOVA. Labeled values without a common letter differ, *P* < 0.05. *HDL-C* High-density-lipoproteins cholesterol, *LDL-C* Low-density-lipoproteins cholesterol, *TG* Triglycerides

### Plasma glucose and insulin

As illustrated in Fig. 2, the concentrations of both glucose and insulin increased within the first 2-h postprandial phase in the control and watercress interventions, but these were lower by consuming the lettuce-containing meal. As well, the maximum values of insulin and the difference between the baseline and their maximum with lettuce consumption were calculated to be less than those of the other two interventions (Table 3). Furthermore, the AUC for glucose was lower in the lettuce intervention compared to the control (*P* = 0.020) and watercress ones (*P* = 0.042). In comparison to the control meal, the AUC for insulin was observed to be lower with the lettuce intervention compared to the control (*P* < 0.05), but no difference was found between the lettuce and watercress interventions (Fig. 2-F).

### Cytokines

There was no significant difference in plasma levels of IL-6 and TNF-α among the three meals (Table 4).

**Table 4:** Plasma inflammatory cytokines before and 4-h after the consumption of control, lettuce or watercress meals in healthy subjects

	Control	Lettuce	Watercress	*P* values		
				Meal	Time	Meal*Time
IL-6 (pg/mL)						
0 h	52.04 ± 10.42	53.55 ± 8.31	62.11 ± 12.45	0.97	0.64	0.10
4 h	58.03 ± 8.64	54.04 ± 8.45	48.41 ± 8.77			
**TNF-α** (pg/mL)						
0 h	24.57 ± 5.35	25.57 ± 5.58	18.84 ± 3.73	0.17	0.17	0.88
4 h	18.13 ± 3.45	21.92 ± 4.68	14.13 ± 2.28			

Values are means ± standard errors. Data were analyzed by repeated-measure ANOVA

*IL-6* Interleukin-6, *TNF-α* Tumor necrosis factor-α

## Discussion

The results of the present study indicate that the consumption of lettuce or watercress with a relatively high-fat meal could not significantly modify the postprandial levels of lipids or inflammatory cytokines. However, in this study, lettuce significantly lowered both postprandial glucose and insulin response levels compared with the control and watercress meals.

Regarding postprandial lipids, plasma TG concentrations after the watercress and lettuce meals were relatively lower than the rate after the control meal at several time points. However, due to relatively large variations in the studied individuals’ responses, no significant differences were found in TG response among the meals. Although no significant differences were observed in AUC for TG, the AUC for HDL-C showed a tendency to be higher after the consumption of watercress or lettuce compared to the control meal. Notably, postprandial TG and HDL metabolism were interconnected [23, 24]. It has been suggested that competition between meal-derived chylomicron remnants and HDL for hepatic lipase may contribute into the prevention of the decreased postprandial in HDL-C [25]. It can be stated that a modification in the affinity of chylomicrons remnants for hepatic lipase after the ingestion of vegetables along with the meal may have resulted both in a tendency for higher postprandial HDL-C concentrations as well as relatively lower postprandial TG levels. Furthermore, following the consumption of a mixed meal, variations in gastric emptying or in the rate of fat digestion in the intestine, could potentially contribute into postprandial plasma TG levels. Since the physical characteristic of food could affect gastric emptying rate [26], so it is possible that both vegetables consumed with the fat-rich meal may consequently influence gastric emptying. In addition, the previous experimental studies have shown that some polyphenols could inhibit pancreatic lipase [27, 28]. Both vegetables, especially watercress [17], were reported as rich in polyphenols and the observed relative lipemic delay may also be attributed to polyphenols present in the consumed vegetables.

In the present study, a delaying effect was observed in the glycemic response to the lettuce consumption as compared to the control and watercress. Accordingly, this finding may be related to the fiber content of meals. It was shown that dietary fiber can change the postprandial glycemic responses by delaying gastric emptying and by hindering the absorption of dietary carbohydrates. Lettuce contains more dietary fibers than watercress (2.4 and 0.5 g of fiber per 100 g of lettuce and watercress, respectively) and this might partly explain the lower glycemic response observed after consuming a meal with lettuce compared to watercress. However, the powder consumed in the control treatment contained 2.4 g of microcrystalline cellulose, which is an insoluble dietary fiber, and despite its wide usage as a control for many dietary fiber studies, it has been shown that it can exert some physiological effects on gastrointestinal physiology and lipid metabolism [29]. Therefore, it can be assumed that other factors may have contributed into the observed effect in the present research. Lettuce has a harder texture than watercress. Zhu et al. in their study showed that the vegetable’s texture itself may play an important role in glucose response [30]. Furthermore, plant bioactive compounds may inhibit the activities of both α-amylase and α-glucosidase. It was demonstrated that the delayed absorption of glucose through the reduction of starch hydrolysis by mildly inhibiting α-amylase and α-glucosidase in the intestine may reduce the rates of dietary carbohydrate digestion and absorption. Interestingly, lactucaxanthin is known as one of the major carotenoids in lettuce [31]. It has been shown that lactucaxanthin may possess some potential anti-diabetic properties, since it could reduce blood glucose level by reducing the activities of both α-amylase and α-glucosidase in the intestine and pancreas of streptozotocin-induced diabetic rats [32]. It should be noted that in some previous studies, changes in glucose and insulin responses have also been monitored during the first hour of intervention (15, 30, and 45 min) [22, 30]. Accordingly, this was a limitation in our study, in a way that the concentrations of plasma variables were not measured before the first hour. It is probable that glucose and insulin peak responses occurred during the first hour, which were missed because the first postprandial blood sampling was performed at 60 min.

In the present study, the consumption of vegetables with meals had no significant effect on the IL-6 and TNF-α concentrations. In healthy individuals, a decrease in inflammatory cytokine levels was reported at 3.5 h after eating a relatively high-fat meal compared to fasting time. However, the consumption of blueberries (which is rich in polyphenols) with the meal has shown no difference in the concentrations of inflammatory cytokines [33]. Another limitation of this study was that the cytokine concentrations were not measured at 4 h post-meal. In a systematic review aimed to characterize the postprandial response of the commonly assessed inflammatory markers after the consumption of a high-fat meal, IL-6 was found to be consistently increased at 4–8 h post-meal [10].

### Study strength and limitations

A strength of the present study was the use of a more realistic test meal with more true-to-life fat levels and energy contents, instead of a very high-fat meal, as the second may not reflect the metabolic state of many individuals in their daily-life [34]. Furthermore, only the male participants were included in the study, which helped in diminishing the potentially confounding effects of female cycles modulating hormone levels on the study parameters. The present study also has some limitations. The sample size was rather small. Furthermore, the study was performed only on healthy young men with normal BMI, which limits the generalization of our findings. Since subjects with obesity may be more prone to postprandial hyperglycemia and hyperlipidemia, so more positive postprandial modulation effects of vegetables may be detected in obese people. As it has been shown, the acute effects of some food ingredients are greater on obese than on normal-weight people [35]. The measurement of postprandial responses to a single meal, as a single meal test, may be misleading, so a second subsequent meal may better explain the effect of the dietary interventions on postprandial TG or glucose excursions [36]. Furthermore, on the days of the meal challenge, physical activity was not similar among the study participants, and about 40% of the participants took short walks (4–5 min) between their blood sampling processes; however, their walking pattern was repeated during all the three meal challenges. Moreover, eating speed of the participants was not recorded as it has been shown that eating speed and eating order could affect the postprandial glycemic response [37]. However, considering that each person’s eating speed probably does not have much day to day variations, the confounding effect of eating speed during each of the three test meals may not have been very significant.

## Conclusion

This study showed that in healthy adults, the addition of lettuce in meals could delay postprandial glycemic response, while watercress results in no change in the overall glycemic response. This finding may be useful for consumers on the choice of each vegetable, considering their effects on postprandial responses. Since lettuce is frequently consumed by people and due to the reason that exaggerated postprandial glycemia is a risk factor for cardiovascular disease, the results of this study may have important applications in clinical practice. Although the present study was conducted on the healthy subjects, the results may have potential health implications for populations at risks of developing diabetes and cardiovascular disease. However, the effects of the consumption of these vegetables on postprandial responses in subjects with cardiometabolic risk factors remain to be investigated yet.

## Data Availability

The datasets analyzed are available from the corresponding author on reasonable request.
